# Oral health-related quality of life in patients with heart failure and left ventricular assist devices—results of a cross-sectional study

**DOI:** 10.1007/s00784-021-03893-w

**Published:** 2021-03-22

**Authors:** Gerhard Schmalz, Christian Binner, Mirjam Eisner, Justus Wagner, Josephine Rast, Tanja Kottmann, Rainer Haak, Sven Lehmann, Michael A. Borger, Jens Garbade, Dirk Ziebolz

**Affiliations:** 1 Department of Cariology, Endodontology and Periodontology, University Leipzig, Liebigstr. 12, D 04103 Leipzig, Germany; 2 University Department for Cardiac Surgery, Leipzig Heart Center, Leipzig, Germany; 3 CRO Dr. med. Kottmann GmbH & Co. KG, Hamm, Germany

**Keywords:** Dental care, Left ventricular assist device, Heart failure, Oral health, Oral-related quality of life

## Abstract

**Objectives:**

The aim of this cross-sectional study was to compare oral health-related quality of life (OHRQoL) of patients with left ventricular assist device (LVAD) and heart failure (HF).

**Material and methods:**

Seventy-four patients with LVAD were recruited from University Department for Cardiac Surgery, Leipzig Heart Center, Germany. A group of 72 patients with HF was composed by matching (age, gender, smoking). The German short form of oral health impact profile (OHIP G14) was applied. Health-related quality of life (HRQoL) was measured by short form 36 survey (SF-36). Dental conditions (decayed-, missing- and filled-teeth [DMF-T]), remaining teeth and periodontal findings were assessed. Statistics: *t*-test, Mann-Whitney *U* test, Kruskal-Wallis test, chi-square or Fisher test, linear regression.

**Results:**

Age, gender, smoking, underlying disease, co-morbidities and oral findings were comparable between groups (*p* > 0.05). OHIP G14 sum score was 3.53 ± 6.82 (LVAD) and 2.92 ± 5.35 (HF; *p* = 0.70), respectively. The scales SF-36 physical functioning (*p* = 0.05) and SF-36 social functioning (*p* < 0.01) were worse in LVAD. In the LVAD group, the DMF-T and remaining teeth negatively correlated with OHIP G14 sum score (*p* < 0.01). In HF patients, positive correlations were found between OHIP G14 and D-T (*p* < 0.01) and remaining teeth (*p* = 0.04). Moreover, DMF-T (*p* = 0.03) and remaining molars/premolars (*p* = 0.02) were negatively correlated with SF-36 scales in HF.

**Conclusions:**

Oral health and OHRQoL was comparable between LVAD and HF; thereby, OHRQoL reflected the clinical oral status.

**Clinical relevance:**

Dental care, with beginning in early stage of HF, should be fostered to preserve teeth and support quality of life before and after LVAD implantation.

## Introduction

For therapy of end-stage heart failure (HF), left ventricular assist devices (LVAD) are becoming increasingly relevant as a definitive substitute for heart transplant or as a bridge to transplantation [1, 2]. Thereby, LVAD are promising in improving cardiac function, survival and quality of life of the patients [2, 3]. In contrast, patients treated with LVAD suffer from a risk of device-related complications as well as complex psychological, emotional and relational complaints, making the real benefit in quality of life still questionable [2, 3].

For these patients, oral health issues might be of particular importance. On the one hand, inflammatory diseases of the oral cavity can be related to a risk of infectious complications [4]. It has been reported that DNA of potentially periodontal pathogens, which are related to periodontal diseases, can be detectable in cardiac tissues [5]. This underlines the potential of these bacteria to cause infectious complications and supports the literature suggesting a relationship between periodontitis and coronary heart diseases [6, 7]. Accordingly, standardized protocols for dental care of LVAD patients before and after implantation are recommendable [8]. The only available previous study found a high prevalence of oral diseases, especially periodontal treatment need and a lack in oral behaviour of LVAD patients, without associations to driveline-related complications [9].

On the other hand, oral health is an influential factor on health-related quality of life (HRQoL) [10]. In this respect, the oral health-related quality of life (OHRQoL) is the sub aspect of HRQoL, which is primarily related to the perception of oral diseases and conditions [11]. As a patient-reported outcome, the assessment of OHRQoL has developed into a mandatory part of evidence-based dentistry [12]. Up until now, no studies are available, which investigate the associations between oral conditions and OHRQoL as well as HRQoL of patients with LVAD. However, this could be of clinical relevance for different reasons. First, potential explanations for insufficient oral status and dental behaviour of these patients might be derivable; for other at-risk patients, e.g. organ transplantation, oral health was shown to be not related to OHRQoL, indicating that their perception of oral health is not in line with the clinical situation [13–15]. Second, the assessment of potential impacts of oral health on OHRQoL and HRQoL could help to develop individual, patient-centred dental care protocols for patients before and after LVAD implantation. For this, a comparison of patients suffering from HF with and without LVAD is needed.

Accordingly, this current study aimed in the comparison of OHRQoL of patients with LVAD and HF. Furthermore, the relationship between oral health and OHRQoL as well as HRQoL was examined. It was hypothesized that both groups show a comparable OHRQoL that is not affected by oral conditions.

## Methods

This current study was designed as a cross-sectional examination to compare the OHRQoL and HRQoL of patients with LVAD and patients suffering from HF. The study has been reviewed and approved by the ethics committee of the Medical Faculty of University of Leipzig (No: 414/16-ek). All patients were informed verbally and in writing and provided their written informed consent.

### Patients

Patients from the University Department for Cardiac Surgery, Leipzig Heart Center, Leipzig, Germany, were included within their routine follow-up appointment between May 2017 and December 2018. The following inclusion criteria were formed for the LVAD group:

- Age of at least 18 years
- Treatment with LVAD, irrespective of time since implantation

Furthermore, the following exclusion criteria did exist:

- Impossibility to undergo full clinical examination due to worse general health status
- Auto-immune diseases (e.g. rheumatoid arthritis)
- Infectious diseases (hepatitis A, B, C, tuberculosis, HIV)
- Pregnancy

Patients, who met these in- and exclusion criteria, were asked for their voluntary participation and were allocated for oral examination, if informed consent was given. Several general and clinical cardiological data, including smoking habits (self-reported as follows: smoker: currently smoking, non-smoker: no smoking at the time of examination), body mass index (BMI, assessed based on the body weight and height at the time point of examination), ejection fraction, underlying heart diseases and co-morbidities, were extracted from the patients’ records. For comparison, a control group of patients suffering from HF from the University Department for Cardiac Surgery, Leipzig Heart Center, Leipzig, Germany, was composed, according to age, gender and smoking habits (matching) of the LVAD group. The exclusion criteria were the same as for the LVAD group.

### Questionnaires

#### Oral health impact profile (OHIP G14)

For the assessment of OHRQoL, the short form of oral health impact profile in German language (OHIPG G14) was used [16–18]. This questionnaire included 14 functional and psychosocial impacts that participants have experienced in the previous month related to their teeth, mouth or dentures. For each question, five answering possibilities, i.e. very often = “4”, fairly often = “3”, occasionally = “2”, hardly ever = “1” and never = “0”, were available. Therefore, a sum score between “0” (all questions answered with “never”) and “56” (all questions answered with “very often”) was achievable. Following the principle of minimal important difference [19], differences in OHIP G14 sum score of at least 2 points were interpreted as clinically relevant. Beside of the OHIP G14 sum score, the two major dimensions “oral function” and “psychosocial impact” were analysed [20].

#### Short form-36 health survey (SF-36)

For the assessment of HRQoL, the short form-36 survey (SF-36) was applied [21]. This questionnaire included a total of 36 items, which can be summarized into the following different scales: physical functioning, role functioning/physical, general health, energy/fatigue, pain, social functioning, emotional well-being and mental well-being. Based on these scales two higher-ordered sum scores, i.e. the physical component summary (PCS) and mental component summary (MCS) measure were determined for further analysis. Data from the scales are presented as raw values (0–100), whereby higher values represent better HRQoL.

### Oral examination

Two experienced and calibrated dentists (kappa > 0.8) performed the oral examinations at the University Department for Cardiac Surgery, Leipzig Heart Center, Leipzig, Germany, under standardized conditions. An antibiotic prophylaxis (2 g amoxicillin or 600 mg clindamycine) according to the recent guidelines was performed for patients with LVAD prior to clinical oral examination [22].

Within a dental examination, the decayed- (D-T), missing- (M-T) and filled-teeth (F-T) index (DMF-T) was assessed visually with mirror and probe in accordance to WHO [23]. Furthermore, the number of remaining teeth (total number of all teeth in the oral cavity), remaining front teeth and remaining molars/premolars was recorded. Within a periodontal examination, periodontal probing depth (PPD) and clinical attachment loss (CAL) was measured with a periodontal probe (PCP 15, Hu-Friedy, Chicago, IL, USA). Thereby, a number of teeth with PPD ≥ 5 mm and CAL ≥ 5 mm were recorded, respectively.

### Statistical analysis

The statistical analysis was performed with SPSS for Windows, version 24.0 (SPSS Inc., USA). The metric variables were tested for their normal-distribution with Kolmogorov-Smirnov test. The analysis of SF-36 was executed with official analysis software (Hogrefe GmbH & Co. KG, Goettingen, Germany). Comparing two independent, normal distributed samples, *t*-test was applied. In case of homogeneity (Levene test), Student’s *t*-test was used. Non-normal distributed samples were analysed with Mann-Whitney *U* test. In comparison of more than two independent, non-normal distributed samples, Kruskal-Wallis test was performed. Categorical data were analysed using chi-square or Fisher test, respectively. For multivariate analysis, a linear regression was conducted. For all applied analyses, a two-sided significance testing was used, whereby the significance level has been set at *p* < 0.05.

## Results

### Patients

A total of 74 patients with LVAD and 72 individuals suffering from HF were included. The mean age, gender distribution and smoking habits were comparable between the two groups (*p* > 0.05). The average time since LVAD was 40.72 ± 22.52 months. The ejection fraction of LVAD patients was significantly lower than in HF patients (*p* < 0.01; Table 1).

**Table 1:** Patient characteristics, significance level: *p* < 0.05

		LVAD (*n* = 74)	HF (*n* = 72)	*p* value
Gender (male in % [*n*])		89.2% [66]	88.9% [64]	0.99
Age in years (mv ± sd)		58.20 ± 9.37	58.20 ± 8.94	0.64
Time since LVAD in months (mv ± sd)		40.72 ± 22.52	–	–
Body mass index (BMI, mv ± sd)		28.95 ± 4.31	28.38 ± 4.95	0.43
Smoking habits % [n]	Non-smoker	91.9% [68]	91.7% [66]	0.99
	Smoker	8.1% [6]	8.3% [6]	
Ejection fraction (mv ± sd)		22.97 ± 6.13	27.97 ± 9.34	**< 0.01**
Underlying heart disease % [*n*]	dcm	48.6% [36]	54.2% [39]	0.19
	icm	48.6% [36]	37.5% [27]	
	Others	2.7% [2]	8.3% [6]	
Co-morbidities % [*n*]	Hypertension	70.3% [52]	77.8% [56]	0.35
	Diabetes mellitus	45.9% [34]	38.9% [28]	0.41
	Renal insufficiency	51.4% [38]	47.2% [34]	0.62
NYHA-classification	I	–	8.3% [6]	–
	II		55.6% [40]	
	III		33.3% [24]	
	IV		2.8% [2]	

Significant results (*p* < 0.05) are highlighted in bold

*LVAD* left ventricular assist device, *HF* heart failure, *mv* mean value, *sd* standard deviation, *dcm*, dilatative cardiomyopathy, *icm* ischemic cardiomyopathy

### Oral examination

Dental parameters (D-T, DMF-T) and periodontal findings (number of teeth with PPD and/or CAL ≥ 5 mm) were comparable between groups (*p* > 0.05). The number of remaining teeth (18.82 ± 9.67 vs. 16.53 ± 8.75; *p* = 0.06) and the number of remaining molars/premolars (9.72 ± 5.56 vs. 8.00 ± 5.41; *p* = 0.06) trended to be higher in LVAD patients (Table 2).

**Table 2:** Results of the dental findings and OHIP G14 scores between groups, significance level: *p* < 0.05

	LVAD (*n* = 74)	HF (*n* = 72)	*p* value
DMF-T (mv ± sd)	17.45 ± 7.66	18.21 ± 7.11	0.57
D-T (mv ± sd)	0.36 ± 1.33	0.50 ± 1.56	0.25
Remaining teeth (mv ± sd)	18.82 ± 9.67	16.53 ± 8.75	0.06
Remaining front teeth (mv ± sd)	9.11 ± 4.39	8.63 ± 3.90	0.13
Remaining molars/premolars (mv ± sd)	9.72 ± 5.56	8.00 ± 5.41	0.06
Number of teeth CAL ≥ 5 mm (mv ± sd)	4.78 ± 5.36	5.07 ± 4.96	0.43
Number of teeth PPD ≥ 5 mm (mv ± sd)	6.72 ± 6.77	5.31 ± 5.42	0.35
OHIP G14 sum (mv ± sd)	3.5 ± 6.8 [0, 0–5.0]	2.9 ± 5.4 [0, 0–4.0]	0.70
Psychosocial impact sum (mv ± sd)	1.9 ± 4.3 [0, 0–1.0]	1.4 ± 3.7 [0, 0–2.0]	0.87
Oral function sum (mv ± sd)	1.1 ± 2.2 [0, 0–1.0]	1.0 ± 1.8 [0, 0–1.0]	0.88

OHIP values are given as mean value ± standard deviation [median; 25th–75th percentile]; significant results (*p* < 0.05) are highlighted in bold

*LVAD* left ventricular assist device, *HF* heart failure, *mv* mean value, *sd* standard deviation, *DMF-T* decayed-, missing- and filled-teeth index, *OHIP* oral health impact profile

### OHIP G14 values

The OHIP G14 sum score was comparable between both groups, showing mean values of 3.5 ± 6.8 (LVAD) and 2.9 ± 5.4 (HF; *p* = 0.70). Moreover, the OHIP G14 dimensions psychosocial impact (*p* = 0.87) and oral function (*p* = 0.88) were comparable between LVAD and HF patients (Table 2). As presented in Table 3, none of the 14 questions from OHIP G14 showed a significant difference between the two groups (Table 3).

**Table 3:** results of the OHIP-G14 questionnaire, significance level: *p* < 0.05

Question [*n*]	Group	Point score					*p* value
		Never (rating 0)	Rarely (rating 1)	Sometimes (rating 2)	Often (rating 3)	Very often (rating 4)	
Trouble pronouncing	LVAD	60	9	5	0	0	0.35
	HF	60	8	2	2	0	
Taste worsened	LVAD	64	6	3	1	0	0.91
	HF	60	8	3	1	0	
Life less satisfying	LVAD	61	8	3	1	1	0.81
	HF	55	12	2	2	1	
Difficult to relax	LVAD	63	6	2	3	0	0.72
	HF	61	7	2	1	1	
Feeling of tension	LVAD	61	10	1	2	0	0.12
	HF	66	3	2	0	1	
Interrupting meals	LVAD	63	8	3	0	0	0.19
	HF	66	6	0	0	0	
Uncomfortable to eat	LVAD	64	6	3	1	0	0.32
	HF	60	8	2	1	0	
Short tempered	LVAD	64	7	2	1	0	0.67
	HF	64	6	1	0	1	
Difficulty performing jobs	LVAD	60	8	3	2	1	0.10
	HF	66	1	4	0	1	
Unable to function	LVAD	63	7	0	3	1	0.14
	HF	68	2	1	0	1	
Embarrassed	LVAD	61	8	3	1	1	0.75
	HF	64	5	2	0	1	
Diet unsatisfactory	LVAD	64	7	1	2	0	0.56
	HF	63	7	1	0	1	
Oral pain	LVAD	59	7	8	0	0	0.33
	HF	57	11	4	0	0	
Sense of uncertainty	LVAD	61	6	6	1	0	0.75
	HF	64	5	2	0	1	

Significant results (*p* < 0.05) are highlighted in bold

*LVAD* left ventricular assist device, *HF* heart failure

### SF-36 values

Two scales of SF-36 were found to be significantly different between the two groups. The scale SF-36 physical functioning was significantly worse in LVAD compared to HF patients (42.1 ± 24.5 vs. 50.0 ± 26.6; *p* = 0.05). Moreover, the scale SF-36 social functioning was worse in the LVAD group (68.6 ± 27.2 vs. 81.4 ± 23.4; *p* < 0.01). Further SF-36 findings are given in Table 4.

**Table 4:** SF-36 between groups, significance level: *p* < 0.05

parameter	LVAD (*n* = 74)	HF (*n* = 72)	*p* value
SF-36 physical functioning (mv ± sd)	42.1 ± 24.5	50.0 ± 26.6	**0.05**
SF-36 role functioning/physical (mv ± sd)	30.1 ± 42.0	27.2 ± 42.2	0.26
SF-36 general health (mv ± sd)	42.5 ± 17.4	45.4 ± 17.7	0.29
SF-36 energy/fatigue (mv ± sd)	46.2 ± 22.1	47.5 ± 18.9	0.62
SF-36 pain (mv ± sd)	75.8 ± 28.8	75.0 ± 26.7	0.61
SF-36 social functioning (mv ± sd)	68.6 ± 27.2	81.4 ± 23.4	**< 0.01**
SF-36 emotional well-being (mv ± sd)	63.1 ± 46.0	70.0 ± 43.0	0.40
SF-36 mental well-being (mv ± sd)	68.7 ± 21.2	69.0 ± 17.6	0.83
Physical compound summary (mv ± sd)	34.9 ± 8.8	36.9 ± 10.3	0.27
Mental compound summary (mv ± sd)	48.8 ± 12.6	50.4 ± 9.3	0.67

Significant results (p<0.05) are highlighted in bold

*LVAD* left ventricular assist device, *HF* heart failure, *mv* mean value, *sd* standard deviation, *SF-36* short form 36 survey

### Correlations between OHIP G14 as well as HRQoL and oral health

In the LVAD group, the DMF-T (*β*: − 1.05, CI95: − 1.57 to − 0.30), number of remaining teeth (*β*: − 1.12, CI95: − 2.54 to − 0.56) and number of remaining molars/premolars (*β*: − 1.26, CI95: − 2.57 to − 0.47) negatively correlated significantly with OHIP G14 sum score (*p* < 0.01; Table 5). In HF patients, positive correlations were found between OHIP G14 and D-T (*β*: 0.60, CI95: 1.45–2.68; *p* < 0.01) as well as the number of remaining teeth (*β*: 0.19, CI95: − 0.01–0.23; *p* = 0.04). Moreover, DMF-T (*β*: − 0.45, CI95: − 1.25 to − 0.06; *p* = 0.03) and number of remaining molars/premolars (*β*: − 0.49, CI95: − 1.73 to − 0.16; *p* = 0.02) were negatively correlated with PCS of SF-36 in HF group. Additionally, a negative correlation between D-T (*β*: − 0.36, CI95: − 3.46 to − 0.82; *p* < 0.01) and MCS of SF-36 was detected in HF patients (Table 6).

**Table 5:** Regression analysis of predictors of OHIP G14 sum score, as well as physical and mental compound summary of SF-36 in patients with LVAD

	OHIP G14		PCS		MCS	
	*β* [CI_95_]	*p* value	*β* [CI_95_]	*p* value	*β* [CI_95_]	*p* value
D-T	0.04 [− 1.25–1.53]	0.76	0.12 [− 0.94–2.54]	0.36	0.04 [− 2.08–2.80]	0.77
DMF-T	− 1.05 [− 1.57 to − 0.30]	**< 0.01**	0.29 [− 0.66–1.32]	0.51	0.54 [− 0.49–2.28]	0.20
Remaining teeth	− 1.12 [− 2.54 to − 0.56]	**< 0.01**	0.27 [− 0.98–1.88]	0.35	0.67 [− 0.62–3.24]	0.26
Remaining molars/premolars	− 1.26 [− 2.57 to − 0.47]	**< 0.01**	0.38 [− 1.05–2.24]	0.47	0.78 [− 0.56–4.06]	0.14

Significant results (p<0.05) are highlighted in bold

*LVAD* left ventricular assist device, *OHIP* oral health impact profile, *SF-36* short form 36 survey, *DMF-T* decayed-, missing- and filled-teeth index

**Table 6:** Regression analysis of predictors of OHIP G14 sum score, as well as physical and mental compound summary of SF-36 in patients with HF

	OHIP G14		PCS		MCS	
	*β* [CI_95_]	*p* value	*β* [CI_95_]	*p* value	*β* [CI_95_]	*p* value
D-T	0.60 [1.45–2.68]	**< 0.01**	− 0.06 [− 3.57–2.84]	0.82	− 0.36 [− 3.46 to − 0.82]	**< 0.01**
DMF-T	0.79 [− 1.18–1.30]	0.92	− 0.45 [− 1.25 to − 0.06]	**0.03**	− 0.46 [− 3.24–2.02]	0.65
Remaining teeth	0.19 [− 0.01–0.23]	**0.04**	− 0.57 [− 3.35–1.98]	0.61	− 0.35 [− 2.84–2.07]	0.76
Remaining molars/premolars	− 0.14 [− 0.89–0.61]	0.71	− 0.49 [− 1.73 to − 0.16]	**0.02**	− 0.18 [− 1.93–1.32]	0.71

Significant results (*p* < 0.05) are highlighted in bold

*HF* heart failure, *OHIP* oral health impact profile, *SF-36* short form 36 survey, *DMF-T* decayed-, missing- and filled-teeth index

## Discussion

### Summary of the main results

Both, oral health and OHRQoL were comparable between LVAD and HF patients. Two scales in SF-36 were worse in LVAD patients. Within the LVAD group, oral conditions were correlated to OHRQoL, while in the HF group correlations were found for both, OHRQoL and HRQoL.

### Comparison with published data

This is the first study, which examined the relationship between oral health and OHRQoL as well as HRQoL in patients with LVAD in comparison to HF patients. Therefore, the comparability to available literature is limited. If data for German general population are considered, a DMF-T of 17.7, a D-T of 0.5 and on average 11.1 missing teeth (means about 17 remaining teeth based on 28 teeth in full dentition) has been reported in fifth German oral health study (DMS V) for patients 65–74 years of age [24]. This appears almost comparable to the current study´s findings for LVAD and HF groups, indicating comparable oral conditions with generally healthy individuals in Germany. Moreover, both groups showed on average more than five teeth with PPD ≥ 5 mm, which argues for a remarkable periodontal treatment need. This is also in line with DMS V for general population [24]. A Chinese cross-sectional study found comparable results, but only examined patients after heart transplantation [25]. In context of oral conditions, a lower number of remaining teeth in LVAD would have been expectable. Recent concepts of dental management prior to LVAD implantation is primarily based on surgical procedures, especially the removal of potential infectious foci by tooth extraction [8, 26, 27]. Remaining teeth were negatively correlated to OHRQoL measurement in the LVAD group. This means that a higher number of remaining teeth is related to lower OHIP G14 sum score, what reflects higher OHRQoL. Therefore, an increased number of remaining teeth, especially molars/premolars appears to have a positive effect on OHRQoL of LVAD patients. This is in line with general literature, showing that tooth loss and the number of functional occlusion pairs (especially molars) are a strong influential factor on OHRQoL [28, 29]. Therefore, dental care protocols for LVAD patients might be more prevention oriented to allow tooth preservation against radical surgical rehabilitation, if possible.

For interpretation of OHRQoL in general, reference values for German population can be consulted. Thereby, values between 0 and 4 points, depending on dentition, can be seen as unaffected OHRQoL [30]. Both, LVAD and HF group are completely within this range. This appears similar to patients after organ transplantation, which also regularly showed an unaffected OHRQoL in comparable studies [13–15]. Accordingly, an unaffected OHRQoL of patients with LVAD and with HF can be concluded from the current study. In contrast to the upper mentioned previous studies on organ transplant recipients [13–15], the examined oral conditions were found to be correlated to OHRQoL in the LVAD as well as HF group. Thereby, the correlation was more strongly in the LVAD group and appears especially relevant regarding remaining teeth.

To interpret the HRQoL of the included individuals, reference values for German patients with heart failure have already been reported [31]. The reference value for PCS is 42.8 ± 9.331, what is slightly higher than in the two groups within the current study. The MCS value of German patients with HF was reported to be 46.6 ± 10.831, what is almost comparable to the current study. Interestingly, LVAD patients in the current study showed worse results in two scales of SF-36, i.e. physical and social functioning. It has been reported that LVAD implantation would lead to improved quality of life [2, 3, 32]. However, a recent systematic review stated that only some aspects of quality of life would be improved after LVAD implantation [33]. The remaining functional and emotional limitations might lead to an impairment of some HRQoL domains, as confirmed in the current study. Thereby it needs to be mentioned, that the HF group was quite heterogeneous regarding the severity of HF (NYHA class). Regardless, oral conditions were found to be negatively correlated with HRQoL in the HF group, indicating that a higher DMF-T and more remaining molars/premolars lead to worse HRQoL. This is somewhat surprising regarding the number of remaining molars/premolars, but might indicate that these remaining teeth cause more complaints if they are still in the oral cavity (e.g. periodontal inflammation, loosening or hypersensitivity). The relation between HRQoL and oral health was not found in the LVAD group. This might indicate that LVAD patients might be primarily affected by LVAD therapy and related complaints in their quality of life. Accordingly, oral conditions only affect OHRQoL in these patients, but not HRQoL. The HF patients, especially if HF is not very severe, perceive affection in both, OHRQoL and HRQoL. However, this remains only speculative and needs to be further evaluated in future studies.

Altogether, the results of the current study indicate several points of clinical relevance. Because of the strong correlation between remaining teeth and OHRQoL in LVAD patients, prevention oriented dental care might be preferable. Furthermore, oral conditions are a predictor of several scales of OHRQoL and HRQoL of patients with HF. In combination with the information that oral diseases, especially periodontitis can be related to morbidity and mortality in patients with severe heart diseases [6, 7], an early dental rehabilitation and consequent dental care appears recommendable. Therefore, special dental care protocols, as demanded in literature [8] should not start immediately before LVAD, but in early stage of HF. A subsequent, individual preventive dental care would stabilize the oral conditions and might avoid the necessity of radical surgical rehabilitation prior to LVAD (and/or heart transplantation). Nevertheless, this needs further evaluation in prospective studies in future. In general, the self-reported oral health (OHRQoL) correlated with objective or clinically assessed oral health status in the current study. Therefore, in this patient population, the self-assessed oral health appear to reflect the oral health status. Already the screening of the OHRQoL with the use of questionnaires, for example, in a cardiology clinic, could detect patients who need to be preferably referred to a dentist. This might be a further approach for improving dental care in these patients.

### Strengths and limitations

This is the first study comparing OHRQoL and HRQoL with regard to oral conditions in patients with LVAD and HF. The applied methods are standardized and valid. The groups were matched and comparable regarding underlying diseases and co-morbidities. Several limitations need to be addressed. Considering the fact that patients with a severe general disease were included, more than 70 patients in each group appear an appropriate sample size; however, no sample size calculation was performed, making the statistical robustness of the findings somewhat questionable. Especially regarding regression analysis, conclusions based on these results must be interpreted with caution. Moreover, the current study was a monocentric, cross-sectional study. Thus, the findings are not representative for Germany and causative conclusions cannot be drawn by this study design. The values of both groups were interpreted with regard to national reference values; for more strong conclusions, a healthy control group might have been recruited, but would not provide relevant additional information for the primary study question. In summary, longitudinal studies with a large sample size should be striven in the future.

## Conclusion

Oral conditions and OHRQoL is comparable between LVAD and HF patients. LVAD patients showed slightly worse HRQoL. Prevention oriented dental care, with beginning in early stage of HF might be recommendable to avoid the necessity of radical surgical rehabilitation before LVAD and should be fostered to preserve teeth and support quality of life in these patients.
